# Sneaking in through the back door

**DOI:** 10.1007/s12471-017-0954-7

**Published:** 2017-02-06

**Authors:** J. J. Piek

**Affiliations:** AMC Heart Center, Academic Medical Center, Amsterdam, The Netherlands

Over recent decades, the numerous coronary revascularisation procedures performed either by percutaneous coronary intervention (PCI), or coronary bypass surgery has led to an increasing number of patients who are no longer eligible for additional revascularisation procedures. This number of patients is expected to rise even further in view of an increase in life expectancy in patients with coronary artery disease.

In a recent issue of the Netherlands Heart Journal, the investigators from the University Medical Center Utrecht report interesting findings on the use of a coronary sinus device in patients with refractory angina in whom there were no options for conventional revascularisation procedures [1]. This device is a balloon expandable stainless steel stent that creates a focal stenosis in the coronary sinus leading to an increase in coronary venous pressure.

The principle of improving myocardial perfusion by elevating coronary venous pressure was introduced as early as 1954 when the coronary sinus was partially ligated in patients with angina pectoris [2]. The publication of the COSIRA trial has rekindled interest in the principle of enhancing coronary venous pressure for relief of angina by means of a stent implantation. This prospective multicentre double-blind sham-controlled trial evaluated coronary sinus stent implantation in patients with refractory angina and demonstrated both symptomatic improvement and an improvement in quality of life over a six-month follow-up period [3]. The study from Abawi et al. [1] evaluated the same coronary sinus stent device as in the COSIRA trial. It is a retrospective analysis of a consecutive series of 23 patients with refractory angina for whom there were no options for coronary revascularisation. In contrast to the COSIRA trial, there were no formal exclusion criteria except the presence of ischaemia related to the perfusion territory of the right coronary artery. Documentation of myocardial ischaemia was a prerequisite and was done either by bicycle stress electrocardiography, dobutamine stress echocardiography, single-photon emission computed tomography (SPECT), or stress magnetic resonance imaging. This is in contrast to the COSIRA trial which only included patients who had undergone positive dobutamine stress echocardiography. The outcome of the present study was anginal status, evaluated by a questionnaire, while evaluation of myocardial ischaemia was not repeated. An improvement in anginal status was documented in 74% of the patients which is in line with the observations of previous non-randomised trials, as well as the COSIRA trial, all of which used the same stent device as in the present study.

The implantation of the balloon expandable stent into the coronary sinus creates a focal stenosis and, as a result, an increase in coronary venous pressure. It is remarkable that none of the aforementioned clinical studies verified this supposed increase in venous pressure by using either fluid-filled catheters or pressure guidewires. There are several postulated mechanisms that may explain the improvement in angina status, although they have not been proven in the clinical setting. First, the increase in venous pressure may lead to the enhancement of collateral blood flow to ischaemic regions [4, 5]. Additionally, the increase of coronary sinus back pressure may lead to a redistribution of flow to the endocardial regions, which are prone to ischaemia, by dilatation of the subendocardial capillaries [5, 6]. Finally, myocardial perfusion may also be improved by neovascularisation as has been shown in a canine experimental model [7].

Coronary back pressure can also be increased intermittently and this modification of the technique has recently been tested in the setting of reperfusion in primary PCI [8]. It has been suggested that this intermittent balloon coronary sinus occlusion may be beneficial due to other mechanisms such as re-opening of the microvasculature, washout of toxic metabolites and activation of vascular regeneration [9]. Being one of the pioneers of intermittent coronary sinus occlusion in the setting of primary PCI, I have always been reluctant to use a flow-limiting device for refractory angina, due to the risk of thrombotic occlusion of this relatively large device in a low pressure environment. It is remarkable that this serious complication did not occur in previous clinical studies or in the present study, where dual antiplatelet therapy (aspirin and clopidogrel) was only given for one month.

The authors are to be commended for conducting this trial and for their tenacity in using this device in patients with refractory angina. Previous clinical trials and the promising results of the present study encourage further exploration of this novel mode of therapy for the increasing number of patients who are not eligible for current revascularisation therapies. The relative simplicity of the implantation techniques and their documented efficacy and safety warrants further evaluation in larger sized trials to verify these interesting findings. We must await the long-term clinical outcome of the patients enrolled in the trials, including their safety, to show if implantation of this coronary sinus device adds to the armamentarium of the coronary interventionalist for the treatment of patients with refractory angina who are no longer candidates for current coronary revascularisation procedures.
